# Characterization of metagenome-assembled genomes from the International Space Station

**DOI:** 10.1186/s40168-023-01545-7

**Published:** 2023-06-01

**Authors:** Nitin K. Singh, Jason M. Wood, Jose Patane, Livia Maria Silva Moura, Jonathan Lombardino, João Carlos Setubal, Kasthuri Venkateswaran

**Affiliations:** 1 Biotechnology and Planetary Protection Group, Jet Propulsion Laboratory, California Institute of Technology, Pasadena, CA 91109 USA; 2 Departamento de Bioquímica, Instituto de Química, Universidade de São Paulo, São Paulo, SP Brazil; 3 Department of Bacteriology, University of Wisconsin–Madison, Madison, WI USA

**Keywords:** Metagenome-assembled genomes, Genome-inferred phenotype, Microgravity, ISS

## Abstract

**Background:**

Several investigations on the microbial diversity and functional properties of the International Space Station (ISS) environment were carried out to understand the influence of spaceflight conditions on the microbial population. However, metagenome-assembled genomes (MAGs) of ISS samples are yet to be generated and subjected to various genomic analyses, including phylogenetic affiliation, predicted functional pathways, antimicrobial resistance, and virulence characteristics.

**Results:**

In total, 46 MAGs were assembled from 21 ISS environmental metagenomes, in which metaSPAdes yielded 20 MAGs and metaWRAP generated 26 MAGs. Among 46 MAGs retrieved, 18 bacterial species were identified, including one novel genus/species combination (*Kalamiella piersonii*) and one novel bacterial species (*Methylobacterium ajmalii*). In addition, four bins exhibited fungal genomes; this is the first-time fungal genomes were assembled from ISS metagenomes. Phylogenetic analyses of five bacterial species showed ISS-specific evolution. The genes pertaining to cell membranes, such as transmembrane transport, cell wall organization, and regulation of cell shape, were enriched. Variations in the antimicrobial-resistant (AMR) and virulence genes of the selected 20 MAGs were characterized to predict the ecology and evolution of biosafety level (BSL) 2 microorganisms in space. Since microbial virulence increases in microgravity, AMR gene sequences of MAGs were compared with genomes of respective ISS isolates and corresponding type strains. Among these 20 MAGs characterized, AMR genes were more prevalent in the *Enterobacter bugandensis* MAG, which has been predominantly isolated from clinical samples. MAGs were further used to analyze if genes involved in AMR and biofilm formation of viable microbes in ISS have variation due to generational evolution in microgravity and radiation pressure.

**Conclusions:**

Comparative analyses of MAGs and whole-genome sequences of related ISS isolates and their type strains were characterized to understand the variation related to the microbial evolution under microgravity. The *Pantoea*/*Kalamiella* strains have the maximum single-nucleotide polymorphisms found within the ISS strains examined. This may suggest that *Pantoea*/*Kalamiella* strains are much more subjective to microgravity changes. The reconstructed genomes will enable researchers to study the evolution of genomes under microgravity and low-dose irradiation compared to the evolution of microbes here on Earth.

Video Abstract

**Supplementary Information:**

The online version contains supplementary material available at 10.1186/s40168-023-01545-7.

## Introduction

Since the publication of the first metagenome-assembled genome (MAG) in 2004 [1], MAGs have been used to confirm genomic data for taxonomic identification of uncultivated microorganisms, metabolic profiling, microbiome dynamics, and host-microbe relationships [2]. When a MAG is assembled, annotation and interpretation of genes are possible, allowing researchers to better understand the metabolic potential of the microbe, including its potential resistance to antibiotics, interactions with other microbes in the microbial community, and association with a host [3]. MAGs have been successfully used to discover uncultivated species [4], candidatus organisms like *Candidatus Amarolinea aalborgensis* gen. nov., sp. nov. [5], and novel genera like *Spiribacter* [6] and *Kalamiella* [7]. MAGs have also been used to recover genomic clusters of secondary metabolites [8], genetic mobility [4], metabolic pathways [9], and in situ replication [10]. Projects retrieving thousands of MAGs have also been documented to understand yet-to-be cultured microbiomes of cow rumens and other environmental samples [11]. In addition, MAGs have revealed new microbial phyla, which have expanded the tree of life [12].

Lowering sequencing cost, curated data availability in public database, and advances in computational biology have made assembling MAGs from complex and extreme environments easy. A higher number of reference genomes makes the binning easier and more reliable. Multiple best practices are available to construct accurate and complete genomes from metagenomes, but high-quality MAGs are still relatively rare. Most MAGs reported in many MAG-related papers have completeness levels between 50 and 60% [13].

Recently, MAGs generated from International Space Station (ISS) environmental metagenomes paved a way to describe a novel genus and species, *Kalamiella piersonii* [7]. We have retrieved four *K. piersonii* MAGs (100% genome sequence identity) from ISS environmental metagenomes that allowed us to identify a cultivated isolate archived from the same samples using gene-specific assays [7]. This “genome to phenome” approach enabled the differentiation of closely related genera *Pantoea* from *Kalamiella* and facilitated the isolation of several ISS strains (*n* = 7) that were archived and previously unidentified [7]. Furthermore, the use of MAGs and whole-genome sequences (WGS) of biosafety level 2 (BSL-2) species, *Klebsiella pneumoniae*, isolated from three consecutive samplings of the ISS at eight defined locations made it possible to track the source of the original BSL-2 strain and understand the succession, persistence, and spread of this opportunistic pathogen detected in the ISS [14].

The objectives of this study were to retrieve near full-length genomes from metagenomes generated from ISS environmental samples and perform in-depth functional and phylogenetic analyses. Among 46 MAGs generated during this study, functional analyses such as antimicrobial resistance (AMR), virulence characteristic, and metabolic traits/stress responses were carried out for 20 prokaryotic MAGs. In addition to the prokaryotic MAGs (~85% completeness), this is the first-time eukaryotic MAGs (~50% completeness) were also generated from ISS metagenomes using the co-assembly strategy.

## Material and methods

### Data source and sample description

Data used for this study were acquired from the National Center for Biotechnology Information (NCBI) Short Read Archive under the bio-project number PRJNA438545. A detailed description of shotgun metagenome sequencing and ISS locations sampled were published elsewhere [14]. In this research communication, we utilized shotgun metagenome reads generated from the propidium monoazide (PMA)-treated samples only [15] to understand whether these MAGs were stemming from the viable and intact cells. However, for eukaryotic analysis, we used shotgun metagenome reads from both PMA and non-PMA-treated samples.

### Metagenome-assembled bacterial genomes

Paired-end 100-bp metagenomic reads were processed with Trimmomatic [16] to remove adapter sequences and low-quality ends, with a minimum Phred score of 20 across the entire length of the read used as a quality cutoff. Reads shorter than 80 bp were removed after trimming. The remaining high-quality reads were subsequently assembled using metaSPAdes [17]. Contigs were binned using Metabat2 [v. 2.11.3] [18]. Recovered genomes were evaluated with CheckM [19], and a recovered genome was considered good with ~85% completeness and at most 10% contamination. Each genome was subsequently annotated with the help of Rapid Annotations using Subsystems Technology (RAST), and taxonomic identifications and phylogenetic affiliations were predicted [20]. In addition to running the procedure above, we also ran the metaWRAP pipeline on the same datasets, using default parameters [21].

In order to test for assemblies suspected to be the same among the 20 metaSPAdes genomes and the set of genomes obtained using metaWRAP for the same set of reads (“meta-set”), we compared both assembly sets using Mash [22] distances. Then, we kept only the best MAG under a Mash distance < = 0.05 (corresponds to average nucleotide identity, *ANI* > = 95%). After gathering the relevant reference genomes and MAGs, Amphora2 [23] was employed to retrieve a protein set composed of 31 universal bacterial markers, which were then aligned by Muscle [24]. Finally, a phylogeny was inferred by maximum likelihood using the concatenated dataset under IQTREE [25] with a concomitant search for the best evolutionary protein model.

For each sample, our final list of bacterial MAGs contains only those MAGs that were considered distinct from one another. When two MAGs were considered “the same” for a given sample, we chose the metaSPAdes version.

### Phylogenetic comparison of the ISS bacterial MAGs obtained by the two methods

MAGs assembled from the ISS dataset by two methods (metaSPAdes metaWRAP) were tested for the phylogenetic clustering within the same species clade. All assemblies from both methods were positioned according to the most exclusive clade in the Bacteria Tree of Life to which individual Genome Taxonomy Database (GTDB) classifications matched (e.g., the most exclusive taxonomic group containing *Staphylococcus*, *Bacillus*, and *Paenibacillus* would be *Bacillales*). After defining such taxonomic groups, a new phylogeny was estimated for each of them. Besides the reconstructed genomes themselves, for each supposed species, we also added three reference genomes from NCBI of the same species (picked sparsely and randomly from its automatically generated species dendrogram tree) while also including two/three other species from the same genus (at least a close species and a not too distant one), each of them being represented by three sparse genomes in their respective species dendrograms. Such an analysis is liberal enough to indicate whether different assemblies from the same reads would indeed cluster together.

### Metagenome-assembled fungal genomes

One of the main focus of this study was assembling eukaryotic genomes from the ISS metagenome. When tools to construct prokaryotic MAGs were used, eukaryotic MAGs were low quality, and results were not satisfactory to identify them as fungal genomes. Additionally, the availability of reference data falls short for eukaryotic genome assembly. To overcome this, we used a completely independent co-assembly-based strategy in the tool ANVIO [18] on the complete ISS dataset using PMA-treated and untreated samples. All the steps were followed as per the step-by-step metagenomic procedure available on the ANVIO website (http://merenlab.org/2016/06/22/anvio-tutorial-v2/). In short, quality filtering was carried out using the script *iu-filter-quality-minoche*. MEGAHIT [26]-based co-assembly was performed on the quality-filtered reads from the 42 samples. Names in the co-assembled fasta file were simplified using the script *anvi-script-reformat-fasta*. A contig database was generated using “anvi-gen-contigs-database.” The contig database was run through hidden Markov models (HMM) based HMMER [27]. NCBI – COG was used to identify the genes in the co-assembly fasta files. Individual profiles were generated for each sample using *anvi-profile* and *anvi-merge* to cluster all the profiles using Euclidean distance and Ward’s linkage algorithm. Final results of co-assembly bins in ANVIO tools were interactively visualized using *anvi-interactive*. All ANVIO results were exported in the summary format for further downstream processing.

Each bin generated in ANVIO profiles was treated as an individual genome, and quality was assessed using CheckM. To establish the taxonomic identification of each bin, they were further subjected to GTDB-Tk (Genome Taxonomy Database Toolkit) [28]. All the bins were compared with 24,706 genomes constituting 8792 validly published bacterial species (https://www.bacterio.net/). GTDB-Tk can only identify prokaryotic genomes; hence, all the bins not defined by the GTDB-Tk were considered as eukaryotic taxa and further manually curated for genomic identification.

Determining the closest fungal species to each MAG was carried out in three steps. First, we used BLASTn to search GenBank, aimed at circumscribing the innermost taxonomic rank quickly (e.g., genus if multiple species within that genus were found across different BLAST hits or family if different genera within the family are found in the BLAST hits). For this search, five different BLASTn searches were performed per MAG. For each such BLASTn search, a random genomic segment of 2000 bp was chosen while also certifying that this segment is within a minimum of 1000 bp from its respective contig end (to minimize possible assembly issues that are more prevalent at contig edges).

Secondly, for those MAGs whose BLAST results were not hitting a single species, phylogenomic analysis was employed with all assemblies/genomes within that innermost rank (as described above) found in either GenBank or Joint Genome Institute (JGI) MycoCosm (with at most three genomes per species). A set of 758 conserved proteins across fungi, available from the BUSCO pipeline [29] (database: fungi_odb10), were sought in every assembly. Because there can be differences in the number of BUSCO genes found per genome, due to variation in assembly completeness, we built a subset of the dataset where each protein is present in at least 1/3 of the MAGs, reference genomes, and two previously chosen outgroups (*Ustilago maydis* and *Dacryopinax primogenitus*) used for proper rooting of the phylogeny. Multiple alignments per gene were carried out using Mafft [30]. Two alternative species tree inference analyses were performed, one IQ, and another inference done by Astral [31], which is based on an amalgamation of quartet trees sampled from each individual gene tree (where each of the 758 gene trees had been previously estimated in IQ-TREE).

Thirdly, Mash distances [22] were computed to confirm the species (either MAGs that went through phylogenomic analyses or MAGs assigned to a single species in the first BLASTn step). According to Gostinčar [32], a Mash distance below 0.04 is sufficient to assign any two fungal genomes to the same species for k-mer sizes between 16 and 22 bp [32].

### Comparative phylogenetic analysis

In order to include a background of Earth-origin genomes to anchor ISS genomes, and therefore pinpoint where in the phylogeny of ISS microorganisms evolved from, we searched in GenBank for genomes of *Staphylococcus aureus*, *S. saprophyticus*, *Klebsiella quasipneumoniae*, *Kalamiella piersonii*, and *Pantoea brenneri*. When available, two genomes of each species per year were retrieved and used in the analysis.

Get_Homologues [33] was used to cluster protein-coding genes into gene families. Unicopy genes (i.e., genes with a single copy in every included genome of the species) were then retrieved to build the phylogenetic trees per species and to further assess amino acid changes. Mafft [30] with default parameters was used to obtain multiple alignments for each gene family. Alignments were then concatenated into a supermatrix using FASconCat [34]. IQ-TREE [25] was used to infer the phylogeny from this supermatrix, using a LG + I + G model and 1000 ultra-fast bootstraps to assess branch support.

In-house python scripts were used to annotate amino acid substitutions and indels (i.e., events of either insertion or deletion of amino acids). Pannzer 2 [35] was used to gather GO information (minimum query and subject cover of 80%, minimum alignment length of 50 aa; other parameters as default) for all genes having amino acid point substitutions that changed hydrophobicity (i.e., from hydrophobic to hydrophilic or vice versa). Such substitutions are more susceptible of being under natural selection, because they have a higher probability of having an impact on the three-dimensional protein structure.

### Comparative functional analysis

Genome assemblies and associated RefSeq annotations for each strain were downloaded from NCBI’s RefSeq database. Due to their exclusion from the RefSeq database, the meta-genome-assembled genomes (MAGs) assemblies and associated annotations were downloaded from their original GenBank accessions. For each of the species of interest in this study, representative type strains were selected for comparison. To compare nucleotide-level identities for each of the analyzed genomes against their respective type strains, BLASTn (−evalue 1e-05) alignments were conducted and visualized with BLAST Ring Image Generator (BRIG), version 0.95 [36]. BLASTn identities were color coded according to the origin of the genome assembly for ISS isolates (purple) or MAGs (blue), with the intensity of their color corresponding to custom percent identity cutoffs (high: 90%, lower: 80%, minimum: 50%). To assess the completeness of each assembly, the open reading frames (ORFs) of 13 housekeeping genes were identified for each species reference genome using keyword searches of the feature_table.txt file included with each assembly: DNA gyrase subunit A (*gyrA*), DNA gyrase subunit B (*gyrB*), 50S ribosomal protein L35 (*rpmI*), 50S ribosomal protein L20 (*rplT*), 30S ribosomal protein S12 (*rpsL*), 30S ribosomal protein S7 (*rpsG*), DNA-directed RNA polymerase subunit beta (*rpoB*), DNA topoisomerase IV subunit A (*parC*), DNA topoisomerase IV subunit B (*parE*), translation initiation factor IF-3 (*infC*), elongation factor Tu (*tuf*), elongation factor G (*fusA*), and cation translocating P-type ATPase (*mgtA*, *zntA*, *actP*, *cadA*, *copB*). Of note, to render MAGs with multiple contigs, BRIG orders each contig into a contiguous assembly to be displayed as a single circular chromosome, causing contig-relative start and end coordinates provided in the assembly feature_table.txt file to not necessarily match the absolute coordinates generated by BRIG. Thus, a custom code was written to transpose the housekeeping genes’ contig-relative start and end coordinates into the absolute coordinates assigned by BRIG. These absolute ORF coordinates were then used as annotations to display on the reference genome on the outermost layer of each BRIG figure. The associated code used to identify these features and convert their coordinates can be found here: https://github.com/jlombo96/MAG_2023_Code.

### Gene-based AMR and biofilm study

Selected genes involved in AMR and biofilm formation were studied. AMR genes were selected based on the abundance in various MAGs, while *E. bugandensis* MAG was chosen for biofilm formation based on the previously reported studies [37].

All AMR gene sequences found in the 20 annotated MAGs were tabulated for comparative analysis. Genes include DNA gyrase and LSU and SSU ribosomal protein units. All identified genes were put into NCBI Nucleotide BLAST (RefSeq), and the top hits with ≥ 98% identity cutoff were compiled in a fasta text file for analysis using MEGA 7 [38]. The genes were aligned using ClusterW, and the Neighbor-Joining algorithm was used to make phylogenetic trees. Nosocomial (hospital/Earth) strains were used for comparison to the MAGs since AMR has been reported to increase under microgravity [14].

The NCBI database was used to derive WGS of *E. bugandensis*. DSMZ and LPSN were used to determine the type strains. Identified strains of *E. bugandensis* were analyzed using the IMG JGI database and used to search for biofilm-forming genes (*pgaA*, *pgaB*, *pgaC*, *pgaD*) and quorum-sensing genes (*LsrK*, *LsrA*, *AI-2 luxS*, *LsrF*, *LsrB*, *LsrC*, *LsrD*, *LsrG*, *LsrR*). All gene sequences were downloaded as a fasta file per strain for downstream alignment analysis.

All downloaded fasta sequences for the biofilm and quorum-sensing genes of all the *E. bugandensis* (*n* = 15 + 1MAG) of interest were used for phylogenetic analysis with the MEGA7 software package. The downloaded fasta sequence for each gene (*n* = 13) in the *E. bugandensis* strains was aligned, conserved sites within that specific gene were observed across all strains versus the ISS isolates, and the variable sites were also observed and quantified for that particular gene.

All areas of interest in the aligned sequences were highlighted and observed for nucleotide differences in each of the 16 strains that are different from that of the EB-247^T^, which is the type strain of the genus. The position of the nucleotide change in the codon was observed to see if it is in the first, second, or third position to determine if it would lead to a synonymous mutation or a new amino acid formation. MEGA7 software using the aligned gene sequence was used to create a neighbor-joining (NJ) phylogenetic tree for each gene with the bootstrap data set at 1000.

## Results and discussion

### Metagenome-assembled bacterial genomes

Out of the 42 ISS metagenomes submitted at NCBI, only PMA-treated metagenomes (*n* = 21) representing the viable/intact cells were used for generating bacterial MAGs. Characteristics of MAGs (*n* = 46) such as genome size (2.6 to 6.6 Mb), completeness, contamination percentage, the average mean coverage, number of scaffolds, and N50 (5 to 670 Kb) were calculated using CheckM, and assembly statistics are summarized in Table 1. Sample collection date, location, relative humidity, radiation exposure, etc. are given in Table 1, and various other details such as materials of the location and partial pressure have already been published [14, 15]. A bacterial MAG was considered acceptable during this study if CheckM completeness was more than 85% and contamination was less than 10%. When metaSPAdes was used, 20 MAGs were recovered, and housekeeping genes were used to confirm the identity, contamination, and completeness of MAGs. In addition, when metaWRAP was used, 26 MAGs were assembled from the same 21 PMA-treated ISS environmental metagenomes. When metaSPAdes and metaWRAP pipelines generated MAGs were compared, a total of 16 one-to-one matches were detected between them, suggesting these two different assembly strategies identified the identical genomes. Among the metaWRAP-assembled genomes, 11 MAGs had smaller genome sizes, which may be due to the more conservative nature of the metaWRAP procedure. The correlation between metaSPAde (20 MAGs) and metaWRAP (26 MAGs) is shown in Supplementary Fig. S1 (*R*^2^ was 0.85).

**Table 1:** Bacterial MAGs recovered from ISS metagenomic samples treated with PMA

Sample^a^	Species identified	NCBI accession no. of type strain	Type strain	ANI with type strain (%)	CheckM completeness (%)	Identified pipeline (MetaSPAdes and metaWRAP)		Location	Temperature	Relative humidity	Radiation measurements (TEPC^b^; µGy; total dose/day session)	Sampling date
F1-2P	*Enterobacter bugandensis*	NZ_LT992502.1	EB-247	98.62	99.37	Yes		Waste and hygiene compartment	22.9	40.20	268	3/4/15
F1-2P	*Klebsiella quasipneumoniae*	CCDF00000000.1	01A030	96.60	99.40	MetaWRAP		Waste and hygiene compartment	22.9	40.20	268	3/4/15
F1-4P	*Staphylococcus aureus*	NZ_CP035101.1	ATCC 12600	98.25	90.81	MetaSPAdes		Surface of the dining table	22.9	40.20	268	3/4/15
F1-5P	*Pantoea brenneri*	MLFN00000000.1	LMG 24534	99.13	100.00	Yes		US node 1, zero-G stowage rack	26.0	40.20	268	3/4/15
F1-7P	*Methylobacterium ajmalii*	GCF_016613415.1	IF7SW-B2	99.98	100.00	MetaWRAP		Overhead 3 panel surface of the Materials Science Research Rack 1	22.2	40.20	268	3/4/15
F1-8P	*Microbacterium aurum*	NZ_BCWI00000000.1	NBRC 15708	95.95	94.11	Yes		Crew quarters-2 bump-out exterior aft wall	22.6	40.20	268	3/4/15
F2-1P	*Acinetobacter pittii*	BBST00000000.1/	DSM 25618	98.26	100.00	MetaSPAdes	Port panel of the cupola		22.8	39.25	380	5/15/15
F2-1P	*Kocuria palustris*	NZ_JAFBCR000000000.1	DSM 11925	98.87	100.00	MetaSPAdes		Port panel of the cupola	22.8	39.25	380	5/15/15
F2-2P	*Staphylococcus saprophyticus*	PPRA00000000.1/	CCUG 38042	99.62	99.44	MetaSPAdes		Waste and hygiene compartment	22.8	39.25	380	5/15/15
F2-5P	*Paenibacillus polymyxa*	NZ_CP049783.1	DSM 36	98.34	99.56	Yes		US node 1, zero-G stowage rack	24.7	39.25	380	5/15/15
F2-5P	*Pantoea brenneri*	MLFN00000000.1	LMG 24534	99.13	100.00	Yes		US node 1, zero-G stowage rack	24.7	39.25	380	5/15/15
F2-7P	*Bacillus velezensis*	LHCC00000000.1/	KCTC 13012	98.05	90.39	MetaWRAP		Overhead-3 panel surface of the Materials Science Research Rack 1	21.8	39.25	380	5/15/15
F2-7P	*Methylobacterium ajmalii*	GCF_016613415.1	IF7SW-B2	99.98	100.00	Yes		Overhead-3 panel surface of the Materials Science Research Rack 1	21.8	39.25	380	5/15/15
F2-7P	*Sphingomonas sanguinis*	BCTY00000000	NBRC 13937	99.71	99.42	Yes		Overhead-3 panel surface of the Materials Science Research Rack 1	21.8	39.25	380	5/15/15
F2-7P	*Staphylococcus saprophyticus*	NC_007350.1	ATCC 15305	99.55	98.89	MetaWRAP		Overhead-3 panel surface of the Materials Science Research Rack 1	21.8	39.25	380	5/15/15
F2-8P	*Methylorubrum extorquens*	NZ_LT962688.1	TK 0001	96.57	98.28	MetaWRAP		Crew quarters-2 bump-out exterior aft wall	22.8	39.25	380	5/15/15
F2-8P	*Paenibacillus polymyxa*	NZ_CP049783.1	DSM 36	98.30	99.85	Yes		Crew quarters-2 bump-out exterior aft wall	22.8	39.25	380	5/15/15
F2-8P	*Staphylococcus aureus*	NZ_CP035101.1	ATCC 12600	97.48	97.46	Yes		Crew quarters-2 bump-out exterior aft wall	22.8	39.25	380	5/15/15
F3-1P	*Kalamiella piersonii*	NZ_RARB00000000.1	IIIF1SW-P2	99.99	89.69	Yes		Port panel of the cupola	20.8	41.25	162	5/5/16
F3-2P	*Cutibacterium acnes*	NZ_CP023676.1	ATCC 6919	99.74	91.36	MetaWRAP		Waste and hygiene compartment	21.9	41.25	162	5/5/16
F3-2P	*Staphylococcus saprophyticus*	NC_007350.1	ATCC 15305	99.33	96.57	Yes		Waste and hygiene compartment	21.9	41.25	162	5/5/16
F3-3P	*Klebsiella pneumoniae*	JOOW00000000	ATCC 13883	96.55	89.66	MetaSPAdes		The foot platform of the Advanced Resistive Exercise Device	21.9	41.25	162	5/5/16
F3-3P	*Klebsiella quasipneumoniae*	NZ_CP012252.1	01A030	96.59	98.47	MetaWRAP		The foot platform of the Advanced Resistive Exercise Device	21.9	41.25	162	5/5/16
F3-3P	*Staphylococcus saprophyticus*	NC_007350.1	ATCC 15305	99.52	82.39	MetaWRAP		The foot platform of the Advanced Resistive Exercise Device	21.9	41.25	162	5/5/16
F3-4P	*Pantoea dispersa*	NZ_SCKT00000000.1	DSM 30073	98.00	99.33	Yes		Surface of the dining table	21.9	41.25	162	5/5/16
F3-4P	*Pantoea dispersa*	NZ_SCKT00000000.1	DSM 30073	97.89	92.92	Yes		Surface of the dining table	21.9	41.25	162	5/5/16
F3-5P	*Kalamiella piersonii*	NZ_RARB00000000.1	IIIF1SW-P2	99.98	88.94	Yes		US node 1, zero-G stowage rack	27.2	41.25	162	5/5/16
F3-7P	*Kalamiella piersonii*	NZ_RARB00000000.1	IIIF1SW-P2	99.98	87.70	Yes		Overhead-3 panel surface of the Materials Science Research Rack 1	21.4	41.25	162	5/5/16
F3-7P	*Klebsiella aerogenes*	NC_015663.1	KCTC 2190	98.52	90.49	MetaWRAP		Overhead-3 panel surface of the Materials Science Research Rack 1	21.4	41.25	162	5/5/16
F3-8P	*Kalamiella piersonii*	NZ_RARB00000000.1	IIIF1SW-P2	99.98	89.38	Yes		Crew quarters-2 bump-out exterior aft wall	20.8	41.25	162	5/5/16

^a^Key to samples, F1, F2, F3; flight, 1, 2, and 3. The numerals after flight numbers denote location from where sampled. P is PMA treated. Descriptions of the locations were published in detail in Singh et al. [37]

^b^*TEPC* US tissue equivalent proportional counter

### Species assignment analysis of ISS bacterial MAGs

Phylogenetic analysis of 30 unique bacterial MAGs of 46 generated showed 18 species (Table 1). The bacterial species identity was based on the average nucleotide index (ANI; > 95%) of MAGs compared with the corresponding type strain. The majority of the MAGs (22 out of 46) belonged to the members of the order Enterobacterales and matched with six established enterobacterial species. In addition, MAGs from spore-forming bacteria (*Bacillus* 1 MAG; *Paenibacillus* 4 MAGs); human skin microbes, e.g., *Staphylococcus* (8 MAGs); and actinobacterial (4 MAGs) members were retrieved. However, 11 MAGs were not identified to any of the genomes of well-established bacterial species. Subsequently, the *gyrB* gene [39] sequences were pulled from the MAGs and screened with sequences of a large number of ISS isolates (*n* = 500 strains) archived in our culture collection. The ISS strains that exhibited the highest *gyrB* sequence similarity (> 95%) were further sequenced for the whole genome and compared with the above 11 MAGs. This “metagenome to phenome” approach has enabled the description of one novel genus/species combination (*Kalamiella piersonii*; 8 MAGs; Singh et al., [7]) and another novel bacterial species, *Methylobacterium ajmalii* (*n* = 3 MAGs; Bijlani et al., 2021). Interestingly, *Sphingomonadaceae* MAGs retrieved from F2-7P samples that matched with yet to be identified *Sphingomonas* sp. K11 strain genome (GenBank no. CP013916.1) also matched with the WGS of three ISS strains isolated from the same location (flight no. 2, location no. 7). These were identified as *Sphingomonas sanguinis*, and functional characteristics were established, and production of plant growth-promoting substances was identified [40].

### Metagenome-assembled fungal genomes

**Fig. 1:**
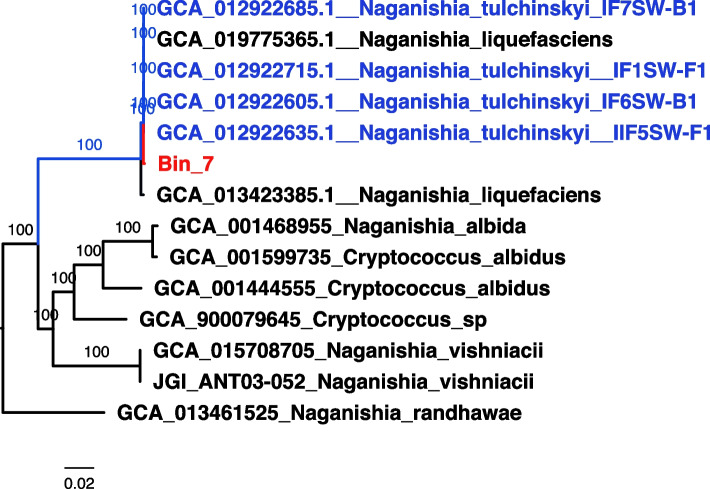
Local phylogeny showing placement of the Bin7 MAG, based on a larger phylogeny embracing 145 *Tremellomycetes* taxa (class where members of the genus *Naganishia* in nested within). Position (in red) relative to its closest clade (in blue) is shown

Conventional tools for MAG assembly are historically not meant for eukaryotic MAG assembly (strategy 1). Additionally, the sequencing depth requirement for eukaryotic MAG assembly is much higher compared to the prokaryotic genomes. We used a co-assembly-based analysis (strategy 2), using the tool ANVIO [18] on the complete ISS dataset containing both the PMA-treated and untreated samples to increase the available data to accommodate eukaryotic MAG assembly. In an effort to make strategy 2 completely independent from strategy 1, the process was rerun using the ANVIO tool from the very initial step of read trimming and filtering. The minimum contig length considered to be included in the data was 1000 bp. The merged profile database that was generated with the minimum contig length of 1000 contained 40,455 contigs, which corresponded to 100% of all contigs, and 100% of all nucleotides found in the contigs database generated during the process. Out of the 84 bins, four bins were classified into the eukaryotic domain. As per the minimum requirement for MAG, completeness should be ≥ 50%, and contamination should be ≤ 10% [41]. Two out of four genomes did not have the required 50% completeness, while the other two were subjected to genome refinement to reduce the redundancy below 10%. After genome evaluation with BUSCO, only four genome bins met the minimal standard for draft MAG, i.e., bins 7, 12, 60, and 73 (Fig. 1).

The four fungal MAGs (Bin7, Bin12, Bin60, and Bin73) could be assigned to the species level by BLASTn searches plus Mash distance comparisons. Bin12 had four (among five) random genomic segments of 2000 bp matching *Rhodotorula mucilaginosa* (ATCC 58901) > 99.9% hit identity, and the remaining segment corresponding *Rhodotorula* sp. (CCFEE 5036). Bin12 Mash distance against the most complete assembly of this species (*R. mucilaginosa* IF1SW-B1, an ISS strain; GCA_013036955.1) was 0.0049, well below the 0.04 Mash distance threshold for k-mer sizes between 16 and 22 bp [32], and therefore confirming it as *R. mucilaginosa* (Table 2). Regarding Bin60, the five BLASTn searches matched *Penicillium chrysogenum* (genome Wisconsin 54-1255; GCA_000710275.1) with percent similarity in the range 98.4–99.95% for the five segments. Mash confirmed the species, with a distance value of 0.007 (Table 2). Regarding Bin73, the five BLASTn searches matched *Papiliotrema laurentii* 5307AH v1.0 with a low Mash distance which confirmed the species as *P. laurentii* and also showed high relationship with the genome of an ISS isolate IF7SW-B5 (GCA_012922625.1; Table 2). Regarding Bin7, BLASTn searches resulted in close matches to members of the *Naganishia* genus. Mash distance calculation against the six closest genomes of this genus (four *Naganishia tulchinskyi* and two *Naganishia liquefaciens*) showed that Bin7 can be assigned to species *N. tulchinskyi* (Table 2); for this bin, a phylogenetic analysis was also carried out confirming the *N. tulchinskyi* classification (Fig. 1). It is interesting to note that the “metagenome to phenome” approach applied for the bacterial MAGs also enabled the description of a novel yeast, *N. tulchinskyi*, from the ISS samples [42].

**Table 2:** Fungal MAGs recovered from ISS metagenomic reads

MAG ID	K-mer size	Query ID	Mash distance	*p*-value	Matching hashes
Bin_12	22	GCA_013036955.1_Rhodotorula_mucilaginosa_IF1SW-B1.fna	0.00485591	0	816/1000
Bin_60	22	GCA_000710275.1_Penicillium_chrysogenum_ASM71027v1_genomic.fna	0.00662984	0	761/1000
Bin_73	22	GCA_012922625.1_Papiliotrema_laurentii_IF7SW-B5.fna	0.00251085	0	898/1000
Bin_7	22	JAAZPV010000089.1_Naganishia_tulchinskyi_strain_IF6SW-B1_scaffold1018_cov224.fna	0.00616116	0	775/1000
Bin_7	22	JAAZPY010000044.1_Naganishia_tulchinskyi_strain_IIF5SW-F1_scaffold102_cov184.fna	0.00616116	0	775/1000
Bin_7	22	JAAZQA010000042.1_Naganishia_tulchinskyi_IF7SW-B1_scaffold100_cov213.fna	0.00616116	0	775/1000
Bin_7	22	JAAZPZ010000100.1_Naganishia_tulchinskyi_strain_IF1SW-F1_scaffold1012_cov89.fna	0.00626056	0	772/1000
Bin_7	22	JACWFY010000001.1_Naganishia_liquefaciens_strain_I2-R1_I2-R1_contig_1.fna	0.00622737	0	773/1000
Bin_7	22	BLZA01000001.1_Naganishia_liquefaciens_N6_DNA.fna	0.0123325	0	616/1000
Bin_7	22	JABRPJ010000001.1_Naganishia_randhawae_strain_eABCC1_contig_1.fna	0.251066	1.2444e-07	2/1000
Bin_7	22	LLJT01000001.1_Naganishia_albida_strain_NT2002_contig1.fna	0.251066	1.27629e-07	2/1000
Bin_7	22	MU158391.1_Naganishia_vishniacii_ANT03-052_unplaced_genomic_scaffold_Nagvi1qcScaffold_1.fna	0.282528	0.000492662	1/1000

**Fig. 2:**
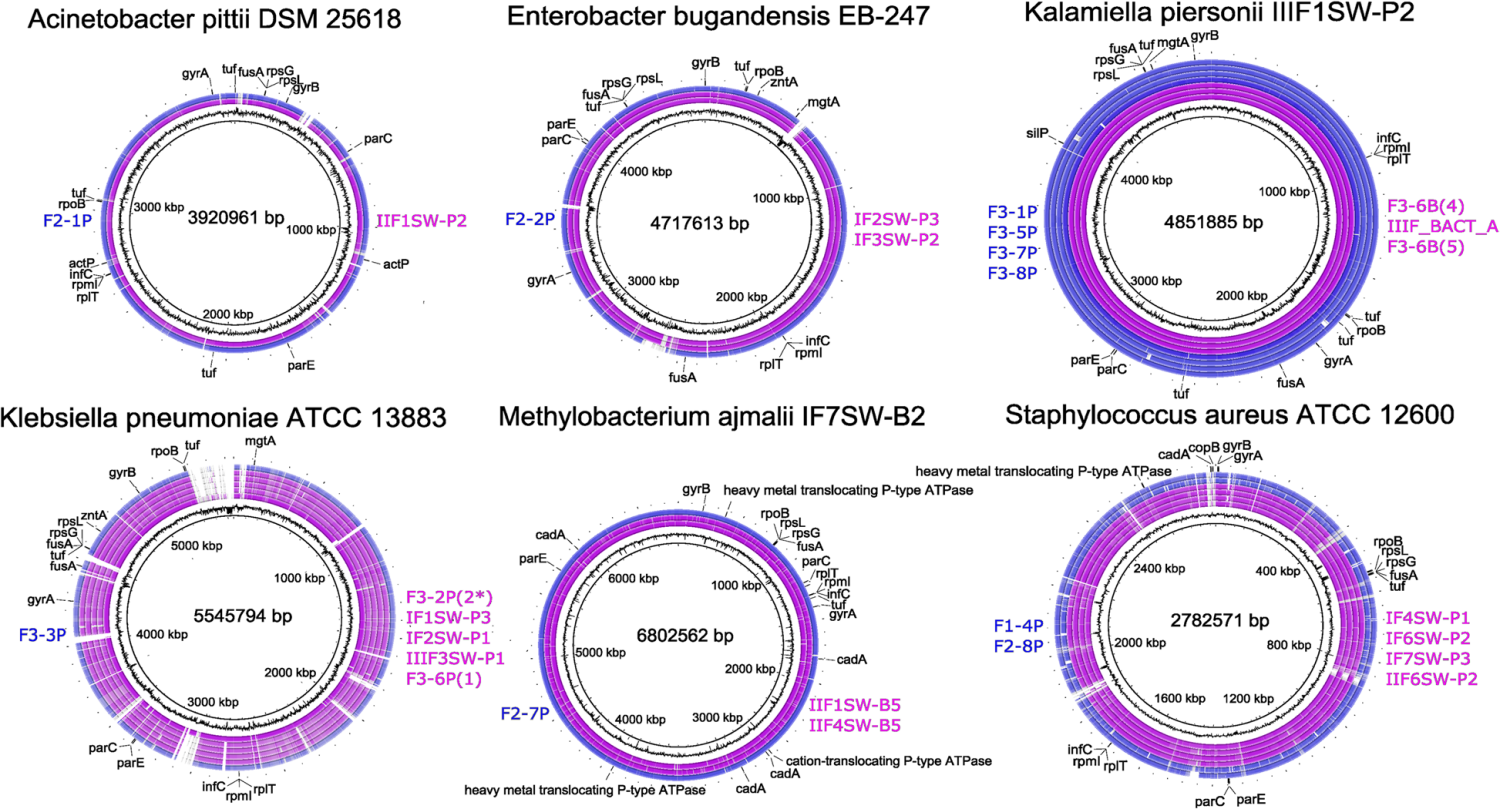
Comparisons of genome assemblies from isolates (purple) and MAGs (blue) against their respective type strains using BRIG. Innermost rings correspond to the pseudo-coordinates of the concatenated reference assemblies and their respective sliding GC content. Ordering of the blast comparisons of each of the assemblies is displayed for the isolates (purple, right) and MAGs (blue, left), ordered from innermost to outermost blast comparison ring. The outermost plot in each figure highlights annotations of relevant markers. Nucleotide identities generated by blastn are color coded for each assembly, with upper identity and lower identity cutoffs at 90% and 80%, respectively, and a minimum *e*-value of 1e-05.

BLAST-based genome comparisons visualized using BRIG for six species are shown in Fig. 2. They are as follows: *Acinetobacter pittii*, *E. bugandensis*, *K. piersonii*, *Klebsiella pneumoniae*, *M. ajmalii*, and *S. aureus*. The BRIG analysis revealed relatively high levels of identity shared across genomes of the ISS isolates and MAGs, when compared against their type strain genomes (Fig. 2). Additionally, patterns of GC content for each bacterial species tested were highly similar to that of their respective type strain.

### Comparative phylogenetic analysis reveals evolution

The possible evolution in the ISS microorganisms was investigated with a careful species-by-species phylogenetic analysis. In this exercise, the following criteria were used: (i) ISS isolates, (ii) ISS MAGs, and (iii) at least one Earth-origin reference genome. The resulting candidate species genome data are shown in Supplementary Table S2, and they belong to *K. piersonii*, *K. quasipneumoniae*, *P. brenneri*, *S. aureus*, and *S. saprophyticus*.

**Fig. 3:**
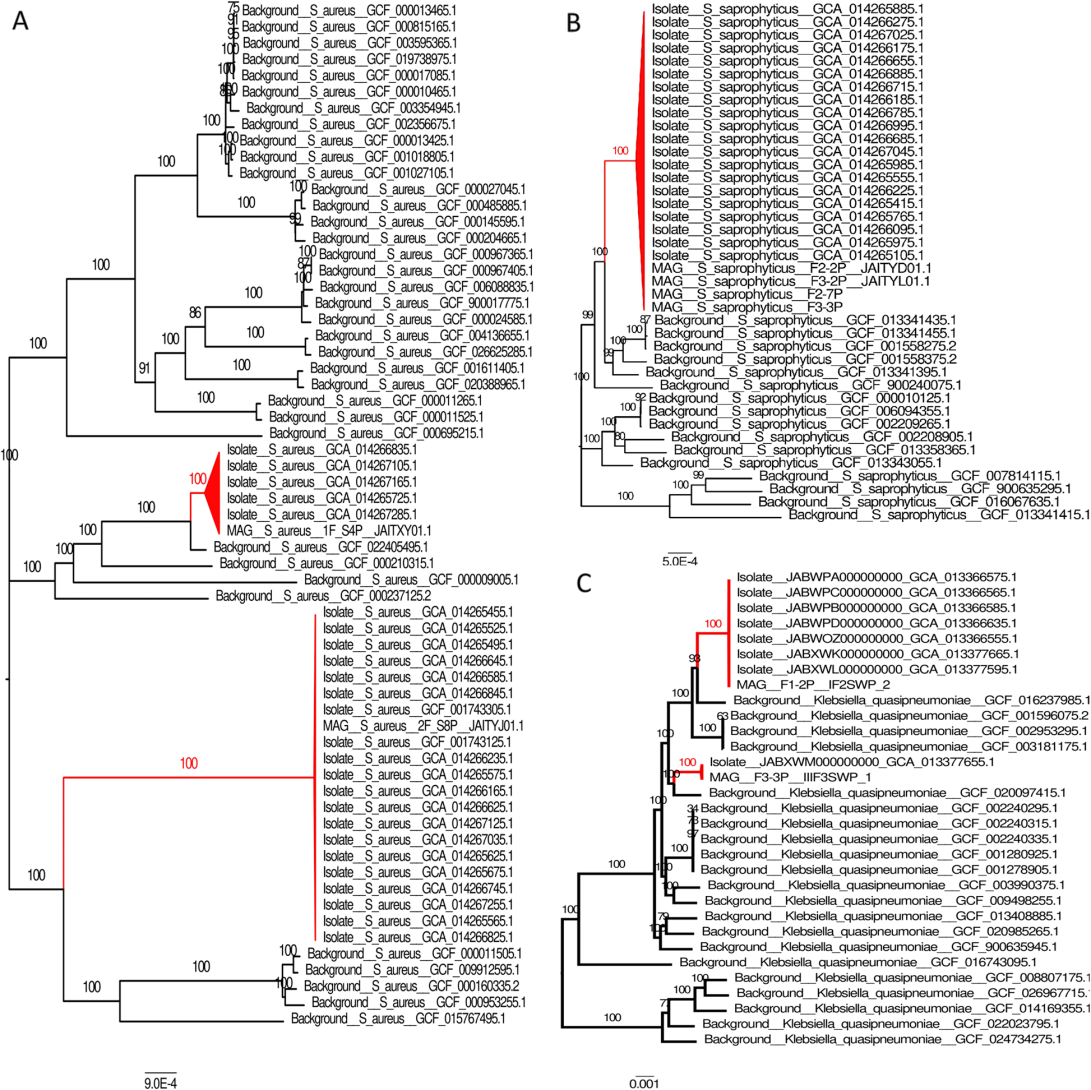
Maximum likelihood phylogenetic trees of **A** *S. aureus*, **B** *S. saprophyticus*, and **C** *Klebsiella quasipneumoniae*. Clades in red contain only ISS genomes

The phylogenetic analyses for those five species showed that ISS genomes (isolates and MAGs) are always monophyletic. This is already suggestive of ISS-specific evolution. For two of those trees (*Kalamiella* and *Pantoea*), there were not enough Earth-origin genomes available (Supplementary Fig. S2). With reference to the *Staphylococcus aureus* and *K. quasipneumoniae* phylogenetic analysis with core genes, the inferred tree contains two ISS clades (Fig. 3 A and C, respectively), suggesting in both cases at least two separate introductions from an Earth source. However, for *S Staphylococcus saprophyticus*, all ISS genomes were placed within a single clade (Fig. 3B).

The single-copy genes specific to either the ISS genomes or the Earth-origin genomes were checked and found none. Furthermore, mutation (indels and substitutions) analyses were carried out that were specific to the ISS clades. The amino acid substitutions that changed a hydrophobic amino acid into a hydrophilic one or vice versa were checked and found many changes. The genes affected by these changes were analyzed in terms of Gene Ontology classifications (Biological Process only; Supplementary Fig. S3). The two ISS-specific clades of the *S. aureus* tree allowed us to compare the two clades in terms of indels. There were 63 indels found to be shared by both clades of *S. aureus*, as opposed to 14 indels specific to clade 1 and 57 specific to clade 2. The GO analysis of the genes affected by the 63 shared indels resulted in 10 GO terms (Supplementary Fig. S4).

Many of the GO terms with high frequency that resulted from these analyses are related to cell membranes, such as transmembrane transport, cell wall organization, and regulation of cell shape. This is consistent with previous reports regarding the effects of microgravity on cellular morphology, proliferation, and adhesion [43]. Taken together, the present results exhibit strong evidence for ISS-specific bacterial evolution.

### Gene-based functional analysis

Variations in the AMR and virulence genes of the selected 20 MAGs have been summarized in Supplemental Table S1. Since multiple studies show that the microbial virulence increases in microgravity [44, 45], AMR gene sequences of MAGs were compared with genomes of type strains and ISS isolates [46, 47]. Among these 20 MAGs characterized, AMR genes were more prevalent in the *E. bugandensis* F1-2P MAG, whereas these were not present in the other MAGs of BSL-2 microorganisms studied during this study.

MAGs were further used to analyze if genes involved in AMR and biofilm formation of viable microbes in the ISS have variation due to generational evolution in microgravity and radiation pressure. Comparative analyses of MAG and WGS of related ISS isolates and their type strains were characterized to understand the variation related to microbial evolution under microgravity. Among 20 MAGs processed, 13 AMR genes were found to be the most prominent among the bacteria (Table 3). These 13 genes were housekeeping genes and have a unique, specialized role determined by their protein type and function. For example, the LSU ribosomal unit is the primary site where protein synthesis occurs in the translation process. Other genes include copper translocating P-type ATPase, DNA gyrase subunits A and B, LSU ribosomal unit 20p and 35p, SSU ribosomal unit 7p, and 12p, DNA-directed RNA polymerase beta subunit, topoisomerase IV subunits A and B, translation initiation factor 3, translation elongation factor Tu (EF-Tu), and translation elongation factor G (EF-G). Functions of genes characterized during this study are given in Supplemental Table S2. Single-nucleotide variation (SNV) among these 13 housekeeping genes in MAGs of six different bacterial species are summarized below.

**Table 3:** Dominant antimicrobial resistance genes identified in ISS-MAGs

		The presence/absence of antimicrobial resistance genes that is as follows:												
Sample	Species identified	Copper-translocating P-type ATPase	DNA gyrase subunit A	DNA gyrase subunit B	DNA-directed RNA polymerase beta subunit	Topoisomerase IV subunit A	Topoisomerase IV subunit B	LSU ribosomal protein L20p	LSU ribosomal protein L35p	SSU ribosomal protein S7p (S5e)	SSU ribosomal protein S7p (S5e)	Translation elongation factor G	Translation elongation factor Tu	Translation initiation factor 3
F1-2P	*Enterobacter bugandensis*	+	+	+	+	+	+	+	+					+
F1-4P	*Staphylococcus aureus*	+	+	+	+	+	++		+	+	+	+	+	+
F1-5P	*Pantoea conspicua*	+	+	+	+	+	+	++	+	+	+	+	+	+
F1-8P	*Microbacterium hominis*	++	++	+	+	+	+			+	+	+	+	
F2-1P	*Acinetobacter pittii*	+	+	+	+	+	+	+	+	+	+	+	+	+
F2-1P	*Kocuria palustris*	+	+	+	+	+	+	+	+	+	+	+	+	+
F2-2P	*Staphylococcus saprophyticus*	+	+	+	+	+	+	+	+	+	+	+	+	+
F2-5P	*Paenibacillus polymyxa*	+	+	+	+	+	+	+	+	+	+	+	+	+
F2-5P	*Pantoea conspicua*	+	+	+	+	+	+	+	+	+	+	+	+	+
F2-7P	*Methylobacterium ajmalii*	+	+	+	+	+	+	+	+	+	+	+	+	+
F2-7P	*Sphingomonas sanguinis*	+	+	+	+	+	+	+	+	+	+	+	+	+
F2-8P	*Paenibacillus polymyxa*	+	+	+	+	+	+	+	+	+	+	+	+	+
F2-8P	*Staphylococcus aureus*	+	+	+	+	+	+	+	+	+	+	+	+	+
F3-1P	*Kalamiella piersonii*	+	+	+	+	+	+	+	+	+	+	+	+	+
F3-2P	*Staphylococcus saprophyticus*	+	+	+	+	+	+	+	+	+	+	+	+	+
F3-3P	*Klebsiella pneumoniae*	+	+	+	+	+	+	+	+	+	+	+	+	+
F3-4P	*Pantoea dispersa*						+	+	+	+	+	+	+	+
F3-5P	*Kalamiella piersonii*	+	+	+	+	+	+	+	+	+	+	+	+	+
F3-7P	*Kalamiella piersonii*	+	+	+	+	+	+	+	+	+	+	+	+	+
F3-8P	*Kalamiella piersonii*	+	+	+	+	+	+	+	+	+	+	+	+	+

Annotated genes identified for various subsystems in 20 selected MAGs are presented in Table 4. The subsystem features include genes responsible for various metabolisms, in which genes responsible for carbohydrates and amino acids metabolism were high. Genes related to motility and chemotaxis were absent in the members of the genera *Acinetobacter*, *Kocuria*, *Staphylococcus*, and *Klebsiella*. In contrast, genes associated with stress response were present in high numbers (90 to 167 genes) within members of family Enterobacteriaceae, whereas such genes were less abundant (19 to 75 genes) with *Staphylococcus* and actinobacterial species. Similarly, sporulation and dormancy genes were mainly present in *Paenibacillus polymyxa*, since they are the only spore-forming bacterium MAG found.

**Table 4:** Annotated genes identified for various subsystems in metagenome-assembled genomes

	Number of annotated genes in the microorganisms that are as follows:									
**Subsystem feature**	***Enterobacter bugandensis***	***Staphylococcus aureus***	***Pantoea conspicua***	***Microbacterium hominis***	***Acinetobacter pittii***	***Kocuria palustris***	***Staphylococcus saprophyticus***	***Paenibacillus polymyxa***	***Pantoea conspicua***	***Methylobacterium ajmalii***
	**F1-2P**	**F1-4P**	**F1-5P**	**F1-8P**	**F2-1P**	**F2-1P**	**F2-2P**	**F2-5P**	**F2-5P**	**F2-7P**
Carbohydrates	614	177	388		213	224	216	351	365	254
Amino acids and derivatives	473	286	392	256	350	290	308	296	377	485
Cofactors, vitamins, prosthetic groups, pigments	268	123	178	120	175	167	104	131	172	210
Protein metabolism	263	94	211	134	207	198	167	182	204	217
Membrane transport	238	61	106	73	107	30	36	56	105	160
RNA metabolism	232	34	59	31	56	24	40	58	59	40
Cell wall and capsule	220	42	42	20	24	10	36	87	42	27
Stress response	167	30	100	19	71	25	33	36	101	75
Regulation and cell signaling	153	34	66	13	46	11	47	27	66	55
Respiration	149	20	117	46	86	35	19	35	104	155
Fatty acids, lipids, and isoprenoids	133	58	72	42	130	85	62	77	71	115
DNA metabolism	130	64	97	62	72	54	71	84	97	95
Nucleosides and nucleotides	113	78	116	87	76	61	76	112	107	93
Virulence, disease, and defense	101	118	74	39	71	38	46	63	73	99
Motility and chemotaxis	96		100					42	100	93
Sulfur metabolism	65	11	34	4	44	20	7	10	34	39
Iron acquisition and metabolism	54	69	28	3	2	4	28	29	28	
Phosphorus metabolism	47	23	43	35	36	25	30	49	43	39
Nitrogen metabolism	44	36	31	7	9	26	10	24	31	15
Metabolism of aromatic compounds	40	3	9	12	76	23	5	4	9	58
Cell division and cell cycle	39	4	8		2	6	5	3	8	2
Miscellaneous	35	11	16	25	41	36	12	15	16	17
Potassium metabolism	30	5	13	4	11	2	2	5	13	10
Dormancy and sporulation	3	8	4	1	2	2	13	38	4	1
*Secondary metabolism*		*5*	*5*	*11*	*7*	*4*	*4*	*6*	*5*	*5*

	Number of annotated genes in the microorganisms that are as follows:									
**Subsystem feature**	***Sphingomonas sanguinis***	***Paenibacillus polymyxa***	***Staphylococcus aureus***	***Kalamiella piersonii***	***Staphylococcus saprophyticus***	***Klebsiella pneumoniae***	***Pantoea dispersa***	***Kalamiella piersonii***	***Kalamiella piersonii***	***Kalamiella piersonii***
	**F2-7P**	**F2-8P**	**F2-8P**	**F3-1P**	**F3-2P**	**F3-3P**	**F3-4P**	**F3-5P**	**F3-7P**	**F3-8P**
Carbohydrates	233	351	177	521	221	498	327	352	350	378
Amino acids and derivatives	247	296	269	557	310	476	398	390	391	421
Cofactors, vitamins, prosthetic groups, pigments	170	129	119	256	98	221	170	175	147	182
Protein metabolism	190	180	143	275	68	196	208	199	217	186
Membrane transport	138	57	44	124	35	115	135	114	96	122
RNA metabolism	38	61	37	88	40	59	61	59	64	66
Cell wall and capsule	26	87	37	60	31	45	51	50	51	54
Stress response	61	37	32	124	33	104	90	97	98	101
Regulation and cell signaling	26	25	28	97	39	91	73	72	71	76
Respiration	116	36	18	141	18	125	101	101	97	110
Fatty acids, lipids, and isoprenoids	64	76	57	72	61	65	102	59	59	61
DNA metabolism	77	85	63	114	73	86	87	82	87	84
Nucleosides and nucleotides	51	111	83	134	80	93	89	95	95	113
Virulence, disease, and defense	120	62	63	80	47	66	53	61	73	69
Motility and chemotaxis	23	43		62			66	57	56	57
Sulfur metabolism	6	11	10	49	7	44	46	37	39	38
Iron acquisition and metabolism	2	29	50	52	25	52	29	38	37	36
Phosphorus metabolism	50	49	20	74	33	45	41	47	46	60
Nitrogen metabolism	11	24	18	44	10	40	25	28	27	28
Metabolism of aromatic compounds	26	4	3	18	5	70	13	17	17	17
Cell division and cell cycle		3	5	9	8	8	8	8	8	8
Miscellaneous	24	15	11	20	11	32	15	16	16	19
Potassium metabolism	10	5	6	17	2	15	15	13	13	13
Dormancy and sporulation	1	38	8	4	8	3	2	4	4	5
*Secondary metabolism*	*5*	*6*	*4*	*5*	*4*	*5*	*4*	*4*	*4*	*4*

#### Copper translocating P-type ATPase

To observe positional sequence variation in the copper translocating P-type ATPase, the *E. bugandensis* F1-2P MAG was compared with the type strain EB-247^T^, which is a nosocomial pathogen isolated from human blood and found sequence variation. Likewise, MAGs of *Pseudomonas brenneri* (F1-5P and F2-5P) and *Pantoea dispersa* (F3-4P) had positional variation compared to their type strains (LMG 24534^T^ and DSM 30073^T^, respectively). In contrast, no sequence variation in the copper translocating P-type ATPase was noticed when analyzing the WGS of the *S. aureus* ATCC12600^T^ type strain, a nosocomial isolate, and *S. aureus* MAGs (F1-4P and F2-8P). Similarly, *K. pneumoniae* MAG F3-3P had no SNVs compared to its type strain ATCC 13883^T^. *Acinetobacter pittii* F2-1P MAG had maximum similarity of copper translocating P-type ATPase sequence with not only its type strain DSM 25618^T^ but also with *Acinetobacter baumannii* DSM 30007^T^ which was isolated from human urine. WGS of the novel species *K. piersonii*, whose type strain IIIF1SW-P2^T^ was also isolated from location no. 1 of the ISS [7], was compared with the MAGs (F3-1P, F3-5P, F3-7P, and F3-8P). This comparison exhibited the same genetic composition, which confirms that the MAGs might have originated from the living cells. In addition, the comparative genomic analysis of the uropathogenic strain of *K. piersonii* strain YU22, isolated from human urine [48], revealed no SNVs.

#### DNA gyrase subunit A

To observe positional sequence variation in the DNA gyrase subunit A, *S. aureus* MAG F2-8P was compared with the genomes of the type strain ATCC 12600^T^, isolated from pleural fluid, and most SNPs were found. However, *S. aureus* MAG F1-4P was highly homologous with the type strain ATCC 12600^T^. In addition, *S. aureus* MAG F2-8P and AR071, a nosocomial strain that is part of the FDA/CDC AMR bank, also showed mutations. Similarly, *E. bugandensis* F1-2P had SNPs found in its counterpart type strain EB-247^T^. *A. pittii* had alignment differences in strain *A. pittii* XJ88, which was found in human sputum which is a mixture of saliva and mucus. *P. brenneri* MAGs (F1-5P and F2-5P) as well as *K. personii* MAGs (F3-5P, F3-8P, and F3-7P) have SNPs with its type strain. The remainder of the *Pantoea* strains was found to have scattered SNPs among Earth homologs. In contrast, *K. quasipneumoniae* MAG F1-2P had no SNPs found with its type strain 01A030^T^, a human blood isolate. *K. pneumoniae* MAG F3-3P had nucleotide differences with NCTC 11357 sequences.

#### DNA gyrase subunit B

*S. aureus* MAGs (F1-4P and F2-8P) had SNPs in *S. aureus* ATCC12600^T^. *E. bugandensis* MAG F1-2P has SNPs found in *E. quasihormaechei* WCHes120003^T^ which was isolated from a human sputum [49] and its type strain as well. *A. pittii* had many scattered SNPs among the Earth homolog. *K. pneumoniae* MAG F3-3P did not have distinct point mutations.

#### DNA-directed RNA polymerase beta subunit

*S. aureus* MAGs (F1-4P and F2-8P) have SNPs found in ATCC 12600, which has already been explained to be a nosocomial strain. *E. bugandensis* F1-2P and Earth homolog *Enterobacter cloacae* complex C45, isolated from a hospital, have point mutations. *K. pneumoniae* F3-3P had SNPs found in *K. pneumoniae* NCTC 9170. *P. dispersa* F3-4P was the only strain that had scattered SNPs in the *Pantoea* species. *Acinetobacter* alignment had SNPs found in *Acinetobacter* sp. genomospecies 3 ATCC 19004 which was isolated from cerebrospinal fluid.

#### LSU ribosomal protein L20p

*S. aureus* F1-4P and F2-8P MAGs had no SNPs with the type strain. Strain *S. aureus* GD1108 which is a hospital strain is the same as that of ISS F1-4P and F2-8P MAGs. *S. aureus* F2-8P ISS strain was very dissimilar to the rest of the *S. aureus* MAG sequences. Both *E. bugandensis* and *Klebsiella* MAGs have no SNPs. The *Pantoea* MAGs have SNPs found in *Plautia stali* (insect) symbiont, *P. vagans* C9-1, and *P. stewartii* DC 283. One SNP was found in strain XJ88 in the *Acinetobacter* (F2-1P) alignment.

#### LSU ribosomal protein L35p

No SNPs were found in all *S. aureus* strains. SNPs were not found in both *Enterobacter* and *Klebsiella* MAGs. *P. brenneri* MAGs (F1A-5P and F2-5P) have no SNPs. *Acinetobacter* had no SNPs found. *K. piersonii* MAGs (F3-1P and F3-7P) have SNPs in *Pantoea* sp. O10 that was isolated from the soil. *P. dispersa* F3-4P has SNPs in *P. rwandensis ND04* (waterfall isolate).

#### SSU ribosomal protein S12p

*S. aureus* MAGs (F1-4P and F2-8P) have SNPs in GD1108. *P. brenneri* MAGs (F1-5P and F2-5P) and *K. personii* MAGs (F3-1P, F3-5P, F3-7P, and F3-8P) have SNPs in strain LMG 24199. *P. dispersa* F3-4P had one SNP found when compared to Earth homolog. *Enterobacter* F1-1P and *Klebsiella* MAGs had no SNPs. The *Acinetobacter* alignment had no SNPs found.

#### SSU ribosomal protein S7p

*S. aureus* MAGs (F1-4P and F2-8P) have SNPs found in their Earth analogs. *P. brenneri* (F1-5P and F2-5P) as well as *K. piersonii* (F3-1P) have SNPs found in LMG24199. *P. dispersa* F3-4P had three SNPs found in the ISS strain. *K. piersonii* MAGs (F3-5P, F3-7P, and F3-8P) had differences found in *Pantoea vagans* FBS135*.* This strain was from a Masson’s pine isolation source. *Enterobacter* F1-1P and *Klebsiella* have no SNPs found. No SNPs were found in *A. pittii*.

#### Topoisomerase IV subunit A

*S. aureus* MAGs (F1-4P and F2-8P) had SNPs found in ATCC 12600. *A. pittii* MAG was extremely different from the rest of the alignment. *E. bugandensis* F1-2P had scattered SNPs. *K. pneumoniae* F3-3P had SNPs found in strain ATCC 700603. *P. brenneri* F2-5P and *K. piersonii* F3-1P had SNPs found in *P. agglomerans* FDAARGOS 160 which was isolated from a human wound isolation source. *P. dispersa* F3-4P and *K. piersonii* F3-7P have no SNPs.

#### Topoisomerase IV subunit B

*S. aureus* F1-4P have no SNPs, but MAG F2-8P had SNPs found in strain AR071 and GD1696. *E. bugandensis* F1-2P MAG was extremely different compared to the rest of the type strain EB-247. *P. brenneri* MAGs (F1-5P and F2-5P) had SNPs found in LMG24199. Other *Pantoea* strains have SNPs primarily found with their respective type strains. *K. piersonii* MAGs (F3-7P and F3-8P) have SNPs found in *Pantoea agglomerans* TH81. *A. pittii* F2-1P had SNPs found in IEC338SC which was isolated from a trachea excretion.

#### Translation initiation factor 3

*S. aureus* MAG F1-4P have had sequences that were very different from its Earth homologs, but there were no SNPs were found in *S. aureus* F2-8P MAG. *K. piersonii* F3-8P had scattered SNPs, whereas MAG F3-7P and *P. dispersa* F3-4P have SNPs in *Plautia stali* symbiont which was collected from the midgut of an insect. All other *Pantoea* MAGs do not have SNPs. The *A. pittii* alignment was found to have SNPs in the strain 201406 which was isolated from human. Both *Enterobacter* and *Klebsiella* species had no nucleotide differences.

#### Translation elongation factor G

*S. aureus* MAGs (F1-4P and F2-8P) have SNPs found in *S. aureus* AR 464. *E. bugandensis* F1-1P has SNPs in *Enterobacter* MBRL1077 which was isolated from a human wound. SNPs were not particular to one *Klebsiella* strain. *K. piersonii* MAGs (F3-1P, F3-5P, F3-7P, and F3-8P) and *P. dispersa* F3-4P have SNPs with their respective type strains. There were no SNPs found in *K. pneumoniae* F3-3P, but SNPs were found in *A. pittii* F2-1P with strain ST220 which was retrieved from sputum. *P. brenneri* F2-5P has no SNPs.

#### Translation elongation factor Tu

*S. aureus* F2-8P had SNPs found in strain AR071, and the rest of the *S. aureus* F1-4P MAG has SNPs in ATCC 12600. *E. bugandensis* F1-2P had scattered SNPs, but *Klebsiella* strains have no SNPs. *P. brenneri* F2-5P and *K. piersonii* MAGs (F3-1P and F3-7P) have SNPs when compared to the type strain. *K. piersonii* F3-5P had SNPs found in *Pantoea stewartii* strain DC283, whereas *K. piersonii* MAG F3-8P did not have any SNPs. *P. dispersa* MAG F3-4P has SNPs in *Pantoea rwandensis* strain ND04. *A. pittii* was extremely dissimilar to its type strain.

Many SNPs found in these 333 alignments have been found in the Earth homologs. The *Pantoea/Kalamiella* strains have the most SNPs found within the ISS strain. This may suggest that *Pantoea/Kalamiella* strains are much more subjective to microgravity changes. More epigenetic and chemical analyses are needed to understand why members of these species are particularly sensitive to these conditions. The difference may also lie in the low percent identity the Earth homologs had. In regards to the *Staphylococcus* strains, ATCC12600 and GD1108 are both nosocomial strains which seem to act differently in each gene. For example, in some genes, ATCC12600 exhibits SNPs, while GD1108 is the only Earth homolog that has the exact same sequence as the ISS strain. Strain GD1108 was isolated from a school child from a prevalence survey in 2011 in Guangzhou, People’s Republic of China [50]. Common SNPs were not found between the ISS and homolog strains within alignments. SNPs in Earth homologs symbolize differences in where the strain was found from. For example, Earth homologs that are found in soil exhibit very different sequences with the ISS strain, therefore explaining the SNPs. In addition, most SNPs have been found in human fluids as well as soil/plants with a few animal excretions. This demonstrates that the ISS strain has some nosocomial/soil background in relation to the rest of the alignment. Strains that are similar/dissimilar to the ISS strain show no common isolation source for the most part. Therefore, more biochemical analyses on the molecular level are needed. In MAG gene analysis (Supplemental Table S1), it was observed that similar genes were responsible for antibiotic resistance and virulence. This common gene-based resistant phenomenon suggests that changes are an adaptation strategy in microbes.

### Genes related to biofilm characteristics

Biofilm forming and quorum-sensing (QS) genes analyzed in this study and their predicted functions are tabulated in Supplemental Table S2. Sequences of all four *E. bungandensis* ISS strains and the MAG (F1-2P) exhibited 100% sequence similarities among them for the genes responsible for biofilm (*pgaABCD*) and QS (*LsrABCD*, *LsrR*, *LsrK*, and *AI-LuxS*) functions. The variable residues (SNPs) of genes related to biofilm formation and QS-related genes in *E. bungandensis* MAG when compared to *E. bugandensis* strains (*n* = 11) isolated from clinical samples are given in Table 5. The comparative sequence analysis of *E. bugandensis* MAG shows that the biofilm and QS genes are highly conserved (< 1% SNPs variation) across most of the clinical strains of *E. bugandensis* (*n* = 10). This pattern is also consistent with the neighbor-joining tree of each one of these genes for all the strains tested and one MAG (data not shown). These analyses confirmed that *E. bugandensis* strains might have hitch-hiked with the healthy crew and landed on ISS surfaces. In contrast, the sequence variation of all biofilm and QS genes of *E. bugandensis* strain MBRL 1077 was highly variable (2.4 to 25%). The average nucleic acid index (ANI) of MBRL 1077 and the *E. bugandensis* type strain EB-247 were ~95%, whereas the ANI was > 99% for the other 10 clinical strains, four ISS isolates, and one MAG. The higher SNPs and lower ANI values of MBRL 1077 strain with all other 14 strains and one MAG suggested that MBRL 1077 might not belong to *E. bugandensis.*

**Table 5:** Differential characteristics of biofilm and quorum sensing-related genes in ISS *Enterobacter bungandensis* (*n* = 4) and F1-2P MAG (*n* = 1) when compared to other related strains

Gene name	Types of residue	Size (bp)	Number of variable residues in genes responsible for biofilm formation and quorum sensing in ISS *Enterobacter bugandensis* strains and MAG when compared to other related strains (GenBank accession no) that are as follows:										
			EB-247 1 NZ_JZZB01000066.1	GN035669	GN02548 NZ_LEDQ01000032.1	GN02283 NZ_LEEJ01000039.1	GN04787 NZ_LVTS01000001.1 NZ	GN03842 NZ_LRCL01000001.1	GN05729 NZ_LRCV01000001.1	MNCRE4 NZ_JZDF01000007.1 NZ_LRCV01000001.1	IIT-BT-08 1 NZ_KI911561.1	MBRL 1077 NZ_CP014280.1 N	153 ECLO NZ_JVSD01000020.1
*zLsrA*	Nucleotide	1488	NA	25	4	33	27	32	27	35	30	156	21
	Amino acid	495	NA	3	0	7	3	4	2	2	2	22	1
*LsrB*	Nucleotide	1002	11	18	18	25	11	30	28	22	19	65	8
	Amino acid	333	1	1	0	1	1	1	2	2	2	7	0
*LsrC*	Nucleotide	1032	23	16	23	90	19	91	21	59	32	85	22
	Amino acid	343	2	4	3	16	2	16	2	8	4	16	2
*LsrD*	Nucleotide	978	1	17	3	67	12	31	13	29	12	83	3
	Amino acid	325	0	3	0	8	2	3	1	2	1	11	0
*LsrF*	Nucleotide	888	31	24	29	17	23	47	25	20	27	66	32
	Amino acid	295	2	2	1	1	1	2	2	1	1	12	1
*LsrG*	Nucleotide	294	11	10	10	21	8	NA	16	98	12	16	5
	Amino acid	97	2	3	1	0	1	NA	3	0	2	2	2
*LsrR*	Nucleotide	972	22	21	9	51	20	23	22	68	34	58	23
	Amino acid	323	2	1	0	5	1	2	2	5	2	1	1
*LsrK*	Nucleotide	1581	28	26	23	75	12	42	26	52	16	132	26
	Amino acid	526	4	3	2	12	1	10	3	6	1	20	5
*AI-luxS*	Nucleotide	576	4	5	3	5	4	6	6	4	5	15	4
	Amino acid	171	0	0	0	0	0	0	0	0	0	0	0
*PgaA*	Nucleotide	2439	30	24	30	31	30	45	29	NA	34	97	22
	Amino acid	812	13	13	12	15	12	23	14	NA	14	35	13
*PgaB*	Nucleotide	1938	31	32	28	30	29	46	13	30	15	83	11
	Amino acid	645	7	9	7	6	8	11	4	8	2	18	1
*PgaC*	Nucleotide	1332	14	13	15	11	16	15	26	11	14	32	14
	Amino acid	443	0	1	2	3	2	2	1	1	1	1	4
*PgaD*	Nucleotide	435	2	2	4	2	2	3	0	4	4	14	1
	Amino acid	144	0	0	0	0	1	0	0	1	2	3	0

NA indicates that the FASTA data for that gene in that specific strain was not found in any database

These SNPs analysis results were also supportive of the function of the genes, e.g., the *AI-luxS* gene had nucleotide mutations; however, none of these nucleotide mutations led to a change of amino acid. This result is interesting because this gene codes for the autoinducer proteins which are very important for carrying signals [51]. In that regard, environmental stress would have no effect on that gene since a change in the gene sequence would not serve to increase the rate of biofilm production and increase the overall amount of biofilm formed, but we can also see that there is a decent amount of amino acid change in the *LsrC* gene which is responsible for importing the autoinducer, and this makes sense because while the autoinducer itself does not need to be changed, the rate at which it is imported to carry information needs to be increased or reduced to accommodate for the change in the amount of biofilm produced. On the other hand, changes in the nucleotide sequence of the *pgaA* and *pgaB* genes, along with the *LsrK* gene, lead to high amino acid change. This could serve as a strong indication of these organisms trying to adjust to the environmental stress because the *pgaA* and *pgaB* are responsible for transporting the PGA essential in the movement of adhesin out of the periplasm to form the biofilm. Also, the *LsrK* is responsible for repressing the *LsrR* repressor, and increasing the function of all three genes will aid in increased signal for biofilm to be formed faster, as well as increase the rate of biofilm formation.

## Conclusion

In summary, good quality bacterial and fungal MAGs were generated from ISS environmental samples, and functional properties were predicted. Furthermore, it was possible to describe novel microbial (two bacterial and one yeast) species via the “metagenome to phenome” approach. The monophyletic phylogeny exhibited by the ISS genomes (both isolates and MAGs) suggested that they shared a single common ancestor. The molecules pertaining to the cell membranes, such as transmembrane transport, cell wall organization, and regulation of cell shape, were in high frequency in ISS genomes demonstrating evidence for ISS-specific bacterial evolution. The common gene-based resistance phenomenon noticed in this study suggests that SNP changes in MAGs and ISS genomes might be an adaptation strategy in AMR and biofilm formation in microbes. Similarly, variations found in the AMR and virulence genes enabled the prediction of the ecology and evolution of microorganisms in space. The maximum SNPs characterized for the ISS *Pantoea/Kalamiella* strains suggested that enterobacterial species are much more subjective to microgravity changes. However, fixation of environmental samples in space for RNAseq approach and/or in situ sequencing in space are warranted to confirm variation related to microbial evolution under microgravity. More studies are needed to unearth whether SNPs seen in ISS MAGs are due to generational evolution in microgravity and radiation pressure.

## Supplementary Information

**Additional file 1.** 

**Additional file 2.**

## Data Availability

The data described in this manuscript can be freely and openly accessed on NCBI Short Read Archive under the bio-project number PRJNA438545.
