# Supplementary material for: Characterization of metagenome-assembled genomes from the International Space Station
Source: Microbiome. 2023 Jun 1;11:125. doi: 10.1186/s40168-023-01545-7 (PMC10233975; doi:10.1186/s40168-023-01545-7)
Supplement: Supplementary file 2 — Additional file 1. [file 40168_2023_1545_MOESM1_ESM.pdf]

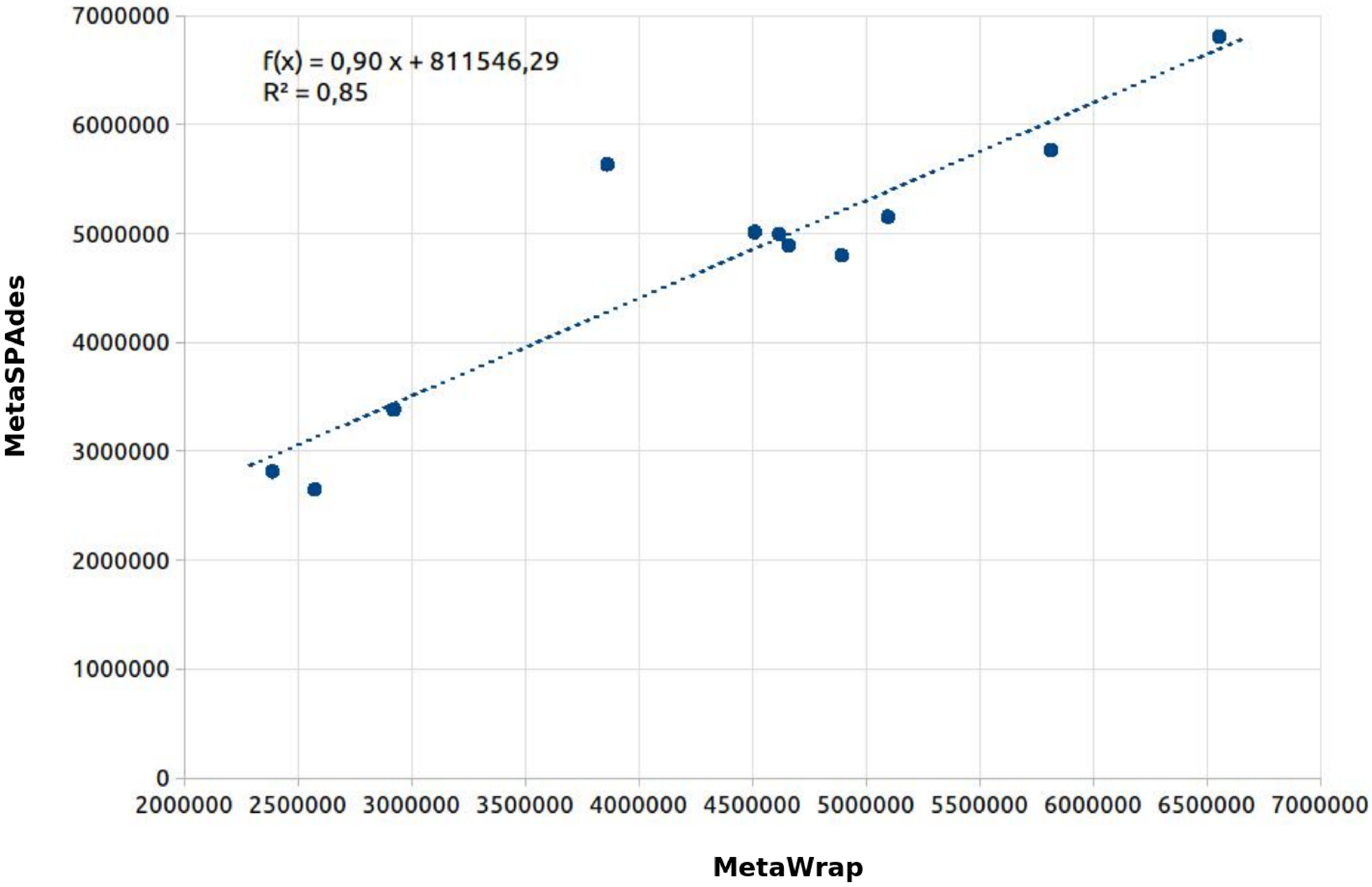

f(x) = 0,90 x + 811546,29
R² = 0,85
MetaSPAdes
MetaWrap
7000000
6000000
5000000
4000000
3000000
2000000
1000000
0
2000000
2500000
3000000
3500000
4000000
4500000
5000000
5500000
6000000
6500000
7000000

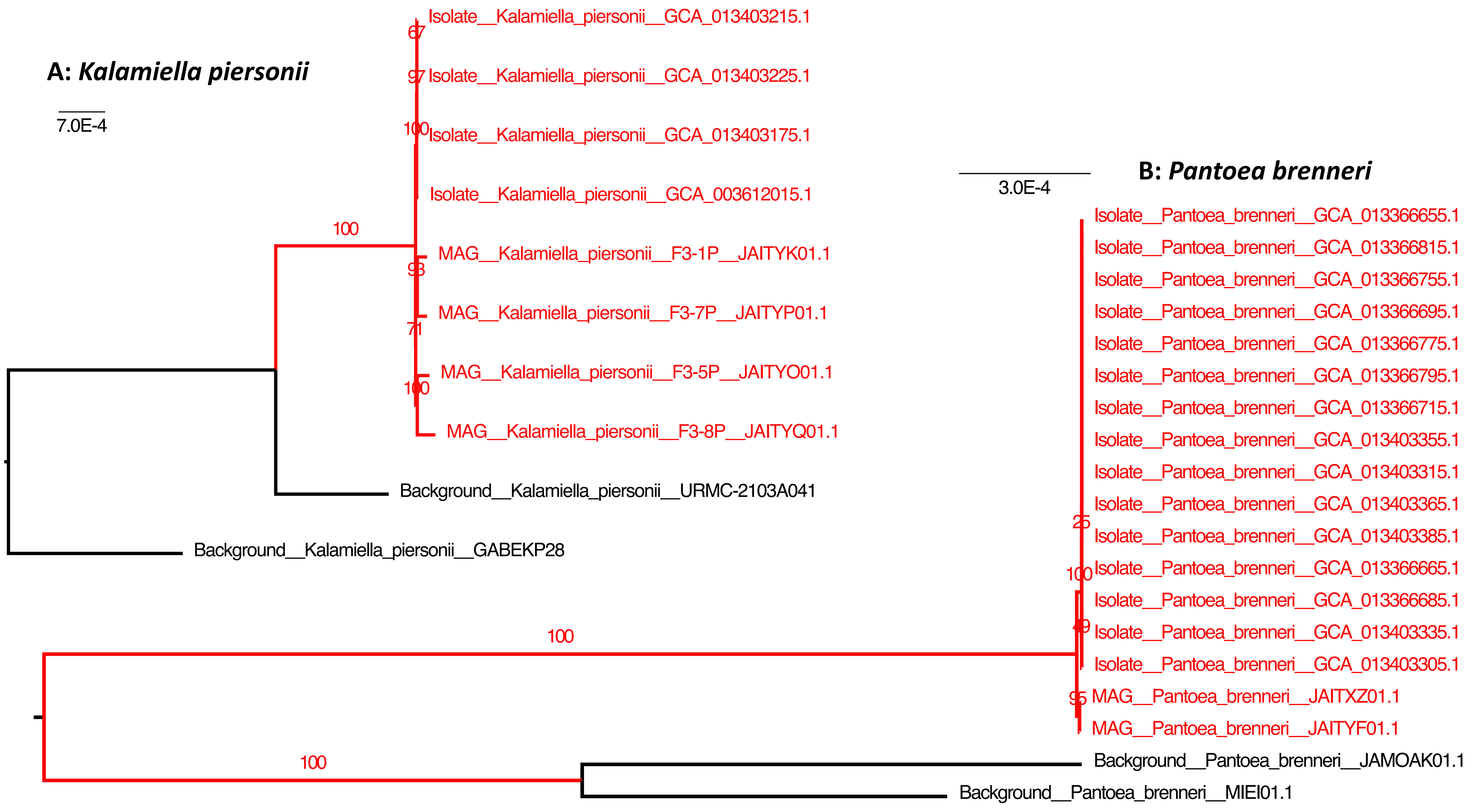
A: Kalamiella piersonii
7.0E-4
Isolate__Kalamiella_piersonii__GCA_013403215.1
Isolate__Kalamiella_piersonii__GCA_013403225.1
Isolate__Kalamiella_piersonii__GCA_013403175.1
Isolate__Kalamiella_piersonii__GCA_003612015.1
MAG__Kalamiella_piersonii__F3-1P__JAITYK01.1
MAG__Kalamiella_piersonii__F3-7P__JAITYP01.1
MAG__Kalamiella_piersonii__F3-5P__JAITYO01.1
MAG__Kalamiella_piersonii__F3-8P__JAITYQ01.1
Background__Kalamiella_piersonii__URMC-2103A041
Background__Kalamiella_piersonii__GABEKP28
67
97
100
100
93
71
100
B: Pantoea brenneri
3.0E-4
Isolate__Pantoea_brenneri__GCA_013366655.1
Isolate__Pantoea_brenneri__GCA_013366815.1
Isolate__Pantoea_brenneri__GCA_013366755.1
Isolate__Pantoea_brenneri__GCA_013366695.1
Isolate__Pantoea_brenneri__GCA_013366775.1
Isolate__Pantoea_brenneri__GCA_013366795.1
Isolate__Pantoea_brenneri__GCA_013366715.1
Isolate__Pantoea_brenneri__GCA_013403355.1
Isolate__Pantoea_brenneri__GCA_013403315.1
Isolate__Pantoea_brenneri__GCA_013403365.1
Isolate__Pantoea_brenneri__GCA_013403385.1
Isolate__Pantoea_brenneri__GCA_013366665.1
Isolate__Pantoea_brenneri__GCA_013366685.1
Isolate__Pantoea_brenneri__GCA_013403335.1
Isolate__Pantoea_brenneri__GCA_013403305.1
MAG__Pantoea_brenneri__JAITXZ01.1
MAG__Pantoea_brenneri__JAITYF01.1
Background__Pantoea_brenneri__JAMOAK01.1
Background__Pantoea_brenneri__MIEI01.1
25
100
49
95
100
100

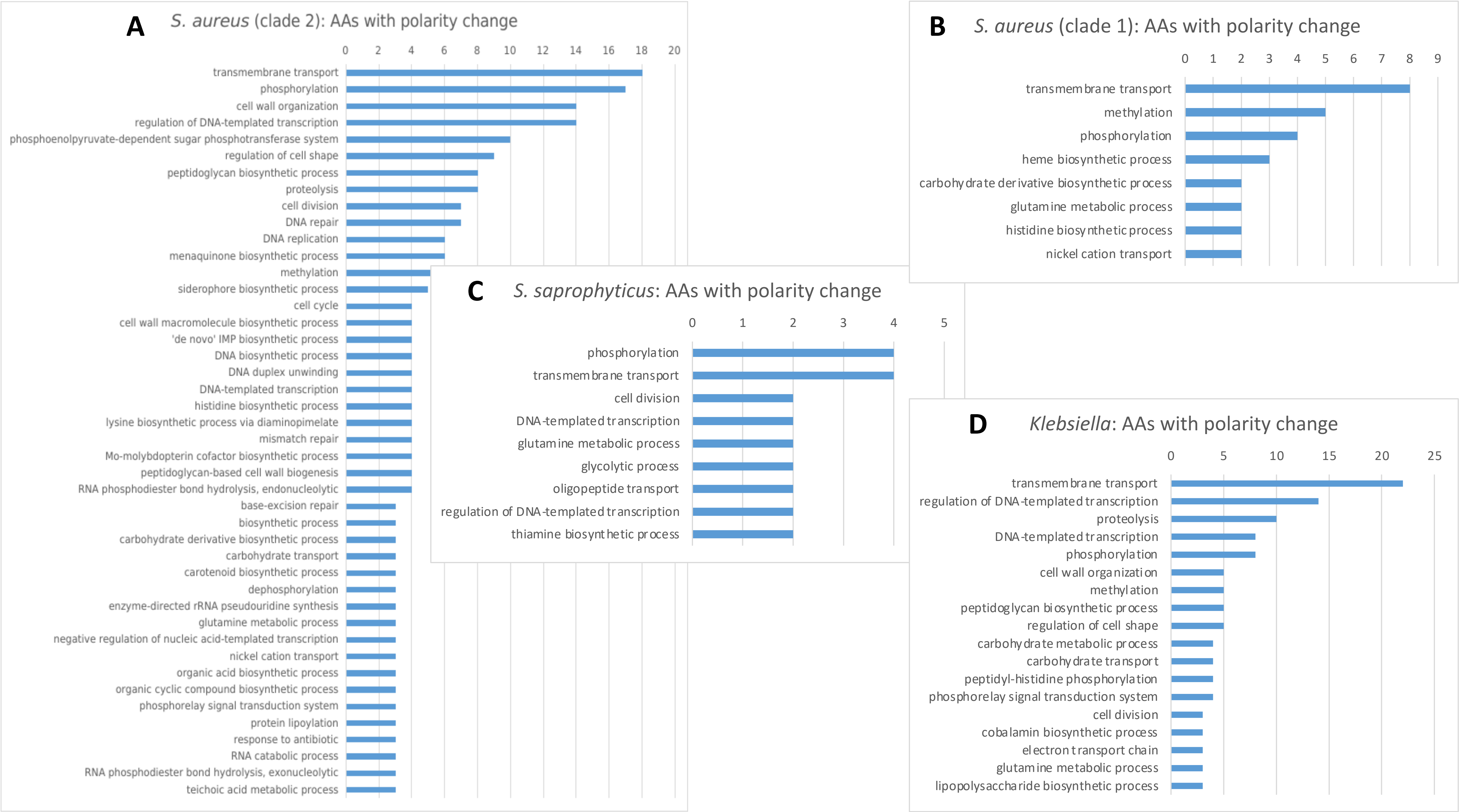
A
S. aureus (clade 2): AAs with polarity change
0 2 4 6 8 10 12 14 16 18 20
transmembrane transport
phosphorylation
cell wall organization
regulation of DNA-templated transcription
phosphoenolpyruvate-dependent sugar phosphotransferase system
regulation of cell shape
peptidoglycan biosynthetic process
proteolysis
cell division
DNA repair
DNA replication
menaquinone biosynthetic process
methylation
siderophore biosynthetic process
cell cycle
cell wall macromolecule biosynthetic process
'de novo' IMP biosynthetic process
DNA biosynthetic process
DNA duplex unwinding
DNA-templated transcription
histidine biosynthetic process
lysine biosynthetic process via diaminopimelate
mismatch repair
Mo-molybdopterin cofactor biosynthetic process
peptidoglycan-based cell wall biogenesis
RNA phosphodiester bond hydrolysis, endonucleolytic
base-excision repair
biosynthetic process
carbohydrate derivative biosynthetic process
carbohydrate transport
carotenoid biosynthetic process
dephosphorylation
enzyme-directed rRNA pseudouridine synthesis
glutamine metabolic process
negative regulation of nucleic acid-templated transcription
nickel cation transport
organic acid biosynthetic process
organic cyclic compound biosynthetic process
phosphorelay signal transduction system
protein lipoylation
response to antibiotic
RNA catabolic process
RNA phosphodiester bond hydrolysis, exonucleolytic
teichoic acid metabolic process
B
S. aureus (clade 1): AAs with polarity change
0 1 2 3 4 5 6 7 8 9
transmembrane transport
methylation
phosphorylation
heme biosynthetic process
carbohydrate derivative biosynthetic process
glutamine metabolic process
histidine biosynthetic process
nickel cation transport
C
S. saprophyticus: AAs with polarity change
0 1 2 3 4 5
phosphorylation
transmembrane transport
cell division
DNA-templated transcription
glutamine metabolic process
glycolytic process
oligopeptide transport
regulation of DNA-templated transcription
thiamine biosynthetic process
D
Klebsiella: AAs with polarity change
0 5 10 15 20 25
transmembrane transport
regulation of DNA-templated transcription
proteolysis
DNA-templated transcription
phosphorylation
cell wall organization
methylation
peptidoglycan biosynthetic process
regulation of cell shape
carbohydrate metabolic process
carbohydrate transport
peptidyl-histidine phosphorylation
phosphorelay signal transduction system
cell division
cobalamin biosynthetic process
electron transport chain
glutamine metabolic process
lipopolysaccharide biosynthetic process

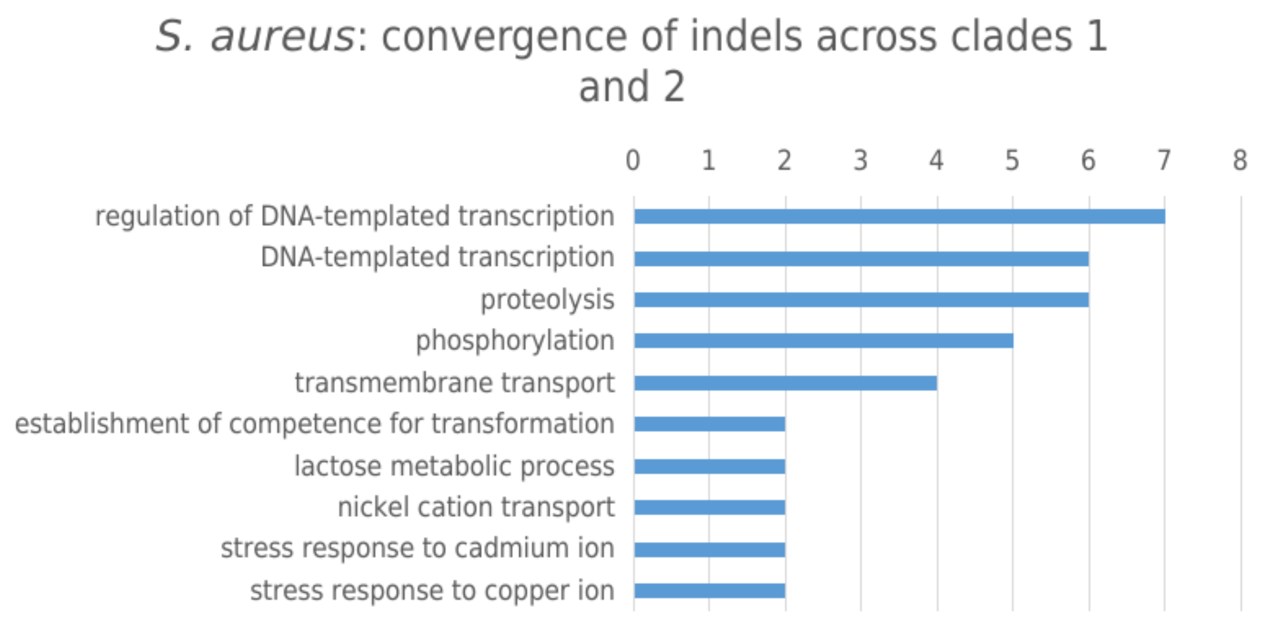
S. aureus: convergence of indels across clades 1 and 2
0
1
2
3
4
5
6
7
8
regulation of DNA-templated transcription
DNA-templated transcription
proteolysis
phosphorylation
transmembrane transport
establishment of competence for transformation
lactose metabolic process
nickel cation transport
stress response to cadmium ion
stress response to copper ion
