# Supplementary material for: Characterization of metagenome-assembled genomes from the International Space Station
Source: Microbiome. 2023 Jun 1;11:125. doi: 10.1186/s40168-023-01545-7 (PMC10233975; doi:10.1186/s40168-023-01545-7)
Supplement: Supplementary file 3 — Additional file 2. [file 40168_2023_1545_MOESM2_ESM.docx]

**Supplementary Table S1**. Genome Data for ISS-specific evolution phylogenetic analysis. The numbers refer to number of genomes available in each category.

| **species** | **ISS MAGs** | **ISS isolates** | **Earth-origin genomes in GenBank** |
| --- | --- | --- | --- |
| *Kalamiella piersonii* | 4 | 4 | 2 |
| *Klebsiella quasipneumoniae* | 2 | 8 | 102 |
| *Pantoea brenneri* | 2 | 15 | 2 |
| *Staphylococcus aureus* | 2 | 25 | 54K |
| *Staphylococcus saprophyticus* | 4 | 20 | 16 |
